# The impact of preoperative venous thromboembolism on patients undergoing TURBT: Perioperative outcomes and healthcare costs from US insurance claims data

**DOI:** 10.1002/bco2.481

**Published:** 2025-01-14

**Authors:** Anas S. Tresh, Francesco Del Giudice, Shufeng Li, Satvir Basran, Ettore De Berardinis, Dalila Carino, Valerio Santarelli, Bernardo Rocco, Maria Chiara Shighinolfi, Roman Mayr, Matteo Ferro, Riccardo Autorino, Gabriele Bignante, Felice Crocetto, Biagio Barone, Renate Pichler, José Daniel Subiela, Jorge Caño Velasco, Marco Moschini, Andrea Mari, Andrea Gallioli, Francesco Soria, Simone Albisinni, Wojciech Krajewski, Jan Łaszkiewicz, Łukasz Nowak, Tomasz Szydełko, Benjamin Challacombe, Rajesh Nair, Benjamin I. Chung

**Affiliations:** ^1^ Department of Urology Stanford University School of Medicine Stanford CA USA; ^2^ Department of Maternal Infant and Urologic Sciences “Sapienza” University of Rome, Policlinico Umberto I Hospital Rome Italy; ^3^ Guy's and St. Thomas' NHS Foundation Trust Guys and St Thomas' Hospital London UK; ^4^ Department of Dermatology Stanford University School of Medicine Stanford CA USA; ^5^ Urologic Unit, ASST Santi Paolo e Carlo La Statale University Milan Italy; ^6^ Department of Urology, St. Josef Medical Center University of Regensburg Regensburg Germany; ^7^ Department of Urology European Institute of Oncology (IEO) IRCCS Milan Italy; ^8^ Department of Urology Rush University Medical Center Chicago IL USA; ^9^ Department of Neurosciences, Reproductive Sciences and Odontostomatology University of Naples Federico II Naples Italy; ^10^ Department of Urology, Comprehensive Cancer Center Innsbruck Medical University of Innsbruck Innsbruck Austria; ^11^ Department of Urology, Hospital Universitario Ramón y Cajal, IRYCIS Universidad de Alcala Madrid Spain; ^12^ Department of Urology Gregorio Marañón University Hospital Madrid Spain; ^13^ Division of Experimental Oncology, Unit of Urology IRCCS Ospedale San Raffaele Milan Italy; ^14^ Urological Robotic Surgery and Renal Transplantation Unit, Careggi Hospital University of Florence Florence Italy; ^15^ Department of Urology, Fundació Puigvert Universitat Autonoma de Barcelona Barcelona Spain; ^16^ Division of Urology, Department of Surgical Sciences, San Giovanni Battista Hospital University of Studies of Torino Turin Italy; ^17^ Unit of Urology, Department of Surgical Sciences Tor Vergata University Rome Italy; ^18^ University Center of Excellence in Urology, Department of Minimally Invasive and Robotic Urology Wroclaw Medical University Wroclaw Poland

**Keywords:** bladder cancer, deep venous thrombosis, health‐related costs, perioperative morbidity, pulmonary embolism, TURBT, venous thromboembolism

## Abstract

**Objectives:**

To assess the impact of a positive history of venous thromboembolism (VTE) on perioperative outcomes, including length of in‐hospital stay, readmission rates, 90‐day postoperative complications, and healthcare costs in bladder cancer (BCa) patients undergoing transurethral resection of bladder tumour (TURBT) in the United States.

**Patients and Methods:**

Patients aged ≥18 years with a BCa diagnosis undergoing TURBT were identified in the Merative® Marketscan® Research de‐identified databases between 2007 and 2021. Multivariable logistic regression adjusted by relevant perioperative confounders was used to investigate the association between diagnosis of VTE before TURBT and 90‐day complication rates, new postoperative VTE events, re‐hospitalization, and total hospital expenditures (2021 US dollars). Sensitivity analyses on VTE severity (pulmonary embolism [PE], deep venous thrombosis [DVT] or superficial thrombophlebitis/phlebitis [SVT]), as well as TURBT extent (minor vs. major) were additionally examined.

**Results:**

In total, 139 800 patients were identified, with 5.3% having preoperative VTE, including DVT (*n* = 3112, 42.20%), PE (*n* = 2046, 27.74%) and SVT (*n* = 2217, 30.06%). A history of preoperative VTE predicted higher rates of any complication (adjusted odds ratio [aOR] 1.28, 95% CI 1.14–1.43) and also higher rates of infectious and haemorrhagic complications. Additionally, preoperative VTE increased the risk of novel VTE events following TURBT (aOR 17.30, 95% CI 16.05–18.65), hospital length of stay (aOR 2.23, 95% CI 1.90–2.62), readmissions (aOR 1.47, 95% CI 1.39–1.56), and hospital associated costs (aOR 1.17, 95% CI 1.12–1.23). DVT and non‐minor TURBT procedures did not increase the risk of any, infectious, or haemorrhagic complications, but other associations were maintained regardless of the severity of VTE (PE, DVT, SVT) or TURBT extent (minor/major).

**Conclusions:**

A history of VTE before undergoing transurethral procedures for BCa is associated with significantly worse perioperative outcomes and higher healthcare costs. These findings may help us to counsel on the risks of the intervention and hopefully improve our ability to mitigate such risks.

## 1 INTRODUCTION

Bladder cancer (BCa) is one of the most common forms of cancer in the United States.^1^ Patients often present with gross haematuria and are then typically taken to the operating room to undergo a transurethral resection of bladder tumour (TURBT), which serves the purpose of diagnosis as well as treatment.^2^, ^3^ Management of bladder cancer is costly given the intensive surveillance schedule and high recurrence rates.^4^ Although the morbidity of this procedure varies, complications generally tend to be minor.^5^ With that being said, the number of BCa cases has been increasing over the years, as well as the number of venous‐thrombotic events (VTE) in these patients following their first 5 years of diagnosis.^6^

VTE consists of both deep vein thrombosis (DVT) and pulmonary embolism (PE).^7^ The association between malignancy and VTE has been well documented in the literature.^8^ This association is of significant importance given that VTE is the leading cause of non‐cancer death in patients who have undergone operations related to their cancer.^9^, ^10^ To our knowledge, there has been no data that has investigated the risks of patients with preoperative VTE undergoing TURBT.

The bulk of the urologic literature has focused on the relationship between intra abdominal surgeries and VTE, with little attention given to endoscopic procedures.^11^, ^12^ This is not surprising given the increased risk of VTE in this population and the morbidity and mortality associated with it.^13^ With that being said, little attention has been given to endoscopic urologic procedures and the risks associated with VTE. Our goal in our study is to assess the health‐related outcomes in patients with BCa and preoperative VTE undergoing TURBT. Our hypothesis is that these patients with known VTE will likely have increased rates of morbidity, hospital costs, and postoperative VTE following intervention with TURBT.

## 2 MATERIALS AND METHODS

### 2.1 Data source

The present research is a retrospective cohort analysis based on administrative insurance claims data from the Merative™ Marketscan® Research Commercial and Medicare databases. The database consists of individual‐level, de‐identified, demographic data, diagnoses, procedures, overall health‐related costs, inpatient and outpatient pharmacy billing claims, and treatments allowing for longitudinal tracking of patients. International Classification of Disease Ninth and Tenth Revisions, Clinical Modification and Procedure Coding System (ICD‐9‐CM, ICD‐10‐CM, ICD‐10‐PCS) codes, Current Procedural Terminology (CPT) and Healthcare Procedure Coding System (HCPCS) codes were used to identify the cohort of interest, treatments and comorbidities. This method has been used in other studies^14^, ^15^, ^16^, ^17^, and given the de‐identified information, the study was deemed exempt from informed consent requirements by the Stanford University Medical Center Institutional Review Board. Data for this project was accessed using the Stanford Center for Population Health Sciences (PHS) Data Core. The PHS Data Core is supported by a National Institutes of Health National Center for Advancing Translational Science Clinical and Translational Science Award (UL1TR003142) and from Internal Stanford funding.

### 2.2 Patients

Using the ICD‐9/10 and CPT diagnosis/treatment codes for BCa and TURBT, data of patients at least 18 years of age who underwent TURBT for BCa between 2007 and 2021 was reviewed in order to create the cohort of interest. Patients selected for analysis were enrolled in the database for at least 3 months before and 3 months following their initial TURBT for primary BC diagnosis recorded in the dataset. The TURBT date was designated as the index date for further assessment. Within this cohort, diagnosis codes were identified and reviewed to ensure the selection of patients with a history of preoperative VTE, defined as PE, lower or upper extremity DVT, or other peripheral/superficial phlebitis/thrombophlebitis (SVT) that was present on admission. The study cohort was further stratified into subgroups based on the presence of VTE before TURBT (‘Preop‐VTE’) versus no history of VTE before TURBT (‘No Preop‐VTE’). It was additionally stratified to minor versus major TURBT. ‘Minor’ TURBT included procedures limited to cystoscopy with biopsy and/or fulguration, differently from ‘major’ which identified procedures being more involved and requiring resection. For each patient, sociodemographic data, including age at index date, gender, US region, and insurance status were initially recorded. Charlson Comorbidity Index (CCI) was calculated according to Charlson and colleagues^18^ and adapted according to Deyo and colleagues.^19^ Also, specific comorbidity prevalence (commonly associated with an increased risk for VTE events) and anticoagulant prophylaxis were recorded. Treatment characteristics included the TURBT procedural data (tumour size, TURBT extent) and perioperative procedures and therapies such as Re‐TURBT and intravesical adjuvant regimens (e.g. chemotherapy and/or BCG). Finally, patients who underwent RC after TURBT, as well as RNU for concomitant UTUC were identified. A flow chart diagram summarizing the analytical steps for the data analysis and inclusion/exclusion criteria is shown in Figure S1, and a detailed list of implemented ICD‐9/10, CPT and HCPCS codes is presented in Table S1.

### 2.3 Outcome ascertainment

Co‐primary endpoints of the study were to ascertain the impact of preoperative VTE on perioperative outcomes and healthcare costs in a cohort of BCa patients undergoing TURBT in the United States. To address these issues, we examined the impact of VTE diagnosis prior to TURBT on 90‐day postoperative complications, 90‐day postoperative novel VTE events, median in‐hospital length of stay, discharge status to self‐care/home vs. any other medical facility, readmission rates, and total healthcare costs associated with the entire TURBT index recovery. The measurement of healthcare costs covered a combination of 90‐day direct hospital costs together with any potentially accountable perioperative interventions, along with adverse events resulting from the index surgery adjusted to 2021 US dollars. A secondary aim of the study was to assess the relative influence of the severity of the VTE event recorded in patients history stratified according to PE versus lower/upper DVT extremity versus SVT on the prespecified outcomes.

### 2.4 Statistical analysis

Continuous variables were reported using means ± standard deviations (SDs) or medians and interquartile ranges (IQR). Categorical variables were presented as counts and percentages (%). The statistical analysis involved using the Chi‐square test for comparison of categorical data, the Student's *t* test for age and the Wilcoxon rank‐sum test for other continuous variables all stratified by preoperative VTE status. Multivariable logistic regression models were implemented to investigate the impact of VTE history on the probability of developing novel VTE events and intra‐ or postoperative surgical complications. Perioperative outcomes, such as length of hospital stay and overall TURBT costs, were tested by applying the median or 3rd quartile distribution as the reference threshold for logistic regression models. Subsequently, logistic regression analyses were replicated after sub‐stratification for the degree of VTE severity (PE, DVT or SVT) and TURBT type (minor or major) in order to explore if the association with perioperative outcomes remained consistent.

All multivariable models were adjusted by clinically relevant variables, including age, CCI score, obesity diagnosis and tumour size. All analyses were two‐sided with, *p* < 0.05 considered significant, and performed using statistical software SAS, version 9.4 (SAS Institute Inc., Cary, NC, USA).

## 3 RESULTS

### 3.1 Study cohort

The study included a cohort of 139 800 patients diagnosed with BCa who underwent TURBT between the years 2007 and 2021 in the United States. Of these, a positive history of VTE before TURBT was present in 7375 (5.3%) patients. Specifically, DVT was the most prevalent type among VTE events (*n* = 3112; 42.20%), followed by PE (*n* = 2046; 27.74%) and SVT (*n* = 2217; 30.06%).

Table 1 shows baseline patient demographic, clinical and hospital characteristics of the whole cohort stratified according to ‘preop‐VTE’ vs. ‘no preop‐VTE’ groups. Preop‐VTE patients exhibited a higher mean age relative to no preop‐VTE individuals (72.2 ± 12.0 years vs. 66.7 ± 13.4 years). A difference in median follow‐up duration between preop‐VTE and no preop‐VTE groups was less than 6 months (median 1.9 years vs. median 2.3 years, respectively). The distribution of patients among the groups was comparable with respect to gender and US geographical region. Comprehensive insurance was more common in the prop‐VTE group (32.5% vs. 22.2%). In addition, preop‐VTE patients were significantly more affected by comorbid conditions (CCI score, median 5 [IQR, 3–8] vs. median 2 [1–4]). Looking closely at the prevalence of specific comorbidities and/or treatment procedures, as expected, preop‐VTE patients had higher rates of obesity (18.7% vs. 9.3%), diabetes (41.4% vs. 26.6%), hypertension (82.6% vs. 61.6%), smoking (20.7% vs. 13.5%) and preoperative anticoagulant prophylaxis (36.4% vs. 9.2%). Both groups were balanced in terms of tumour size, TURBT extent and perioperative therapies. Finally, a similar proportion of patients in the groups underwent radical cystectomy (RC) and radical nephroureterectomy (RNU).

**TABLE 1:** Baseline patient demographic, clinical and hospital characteristics of the final cohort of the study according to VTE history prior to TURBT (preop‐VTE vs. no preop‐VTE).

	Preop‐VTE	No Preop‐VTE	*p* Value
Number (%)	7375 (5.3)	132 425 (94.7)	
VTE severity (%)			
DVT	3112 (42.2)	NA	
PE	2046 (27.7)	NA	
SVT	2217 (30.1)	NA	
Age, mean (SD)	72.2 (12.0)	66.7 (13.4)	<0.0001
1st quartile	953 (12.9)	34 354 (25.9)	<0.0001
2nd quartile	1429 (19.4)	33 385 (25.2)	
3rd quartile	2164 (29.3)	32 246 (24.4)	
4th quartile	2829 (38.4)	32 440 (24.5)	
Follow‐up years, median (IQR)	1.9 (0.9–3.7)	2.3 (1.0–4.5)	<0.0001
Gender, *n* (%)			0.3359
Male	5195 (70.4)	92 582 (69.9)	
Female	2180 (29.6)	39 843 (30.1)	
US region, *n* (%)			<0.0001
Northeast	1716 (23.3)	31 560 (23.8)	
North Central	2490 (33.8)	35 345 (26.7)	
South	2151 (29.2)	44 046 (33.3)	
West	922 (12.5)	18 793 (14.2)	
Unknown	96 (1.3)	2681 (2.0)	
Data source, *n* (%)			<0.0001
Fee for Service	1911 (25.9)	56 120 (42.4)	
Encounter	218 (3.0)	7179 (5.4)	
Medicare	4704 (63.8)	61 025 (46.1)	
Medicare Encounter	534 (7.2)	7955 (6.0)	
Insurance type, *n* (%)			<0.0001
Comprehensive	2399 (32.5)	29 439 (22.2)	
HMO	713 (9.7)	14 307 (10.8)	
PPO	3424 (46.4)	67 898 (51.3)	
Other	839 (11.4)	20 781 (15.7)	
CCI, median (IQR)	5 (3–8)	2 (1–4)	<0.0001
0–1	729 (9.9)	38 196 (28.8)	<0.0001
2–4	2621 (35.5)	67 487 (51.0)	
≥5	4025 (54.6)	26 742 (20.2)	
Prevalent comorbidities, *n* (%)			
Obesity	1377 (18.7)	12 359 (9.3)	<0.0001
Diabetes	3054 (41.4)	35 182 (26.6)	<0.0001
Hypertension	6088 (82.6)	81 502 (61.6)	<0.0001
Renal failure	2568 (34.8)	18 504 (14.0)	<0.0001
Smoking	1525 (20.7)	17 888 (13.5)	<0.0001
Infectious	3839 (52.05)	38 143 (28.8)	<0.0001
Hematologic	4353 (59.0)	32 453 (24.5)	<0.0001
Anticoagulant prophylaxis, *n* (%)			
90 days before TURBT	2681 (36.4)	12 171 (9.2)	<0.0001
90 days after TURBT	2534 (34.4)	12 645 (9.6)	<0.0001
Tumour size, *n* (%)	*n* = 5188	*n* = 92 171	0.0065
Small (≤ 2 cm)	1199 (23.1)	23 108 (25.1)	
Medium (2–5 cm)	2098 (40.4)	36 297 (39.4)	
Large (> 5 cm)	1891 (36.5)	32 766 (35.6)	
Minor TURBT, *n* (%)			0.0006
No	2650 (35.9)	45 005 (34.0)	
Yes	4725 (64.1)	87 420 (66.0)	
Re‐TURBT < 90 days from TURBT, *n* (%)	1272 (17.3)	23 565 (17.8)	0.2312
Time from TURBT to Re‐TURBT, weeks, median (IQR)	4.1 (1.4–7.1)	4.1 (1.3–7.1)	0.3745
Intravesical chemotherapy, *n* (%)	2246 (30.5)	44 540 (33.6)	<0.0001
PIC (≤ 14 days after TURBT)	554 (24.7)	11 909 (26.7)	0.1780
Adjuvant CHT (> 90 days after TURBT)	686 (30.5)	13 380 (30.0)	
Intravesical BCG after TURBT, *n* (%)	1490 (20.2)	30 255 (22.9)	<0.0001
Time from TURBT to intravesical BCG, months, median (IQR)	2.0 (1.2–4.8)	1.9 (1.1–5.1)	0.8114
RC after TURBT (i.e. primary MIBC), *n* (%)	470 (6.4)	10 158 (7.7)	<0.0001
Time from TURBT to RC, months, median (IQR)	3.3 (1.6–8.6)	3.9 (1.6–7.5)	0.5622
Concomitant UTUC, *n* (%)	202 (2.7)	2616 (2.0)	<0.0001
RNU before TURBT, *n* (%)	162 (2.2)	1146 (0.9)	<0.0001
Time to RNU before TURBT, months, median (IQR)	8.9 (4.9–20.8)	7.2 (4.2–14.2)	0.0199
RNU after TURBT, *n* (%)	160 (2.2)	2773 (2.0)	0.6598
Time to RNU after TURBT, months, median (IQR)	2.7 (1.4–8.2)	3.5 (1.3–14.6)	0.1340

Abbreviations: BCG, *Bacillus Calmette–Guérin*; CCI, Charlson Comorbidity Index; CHT, chemotherapy; DVT, deep venous thrombosis; HMO, Health Maintenance Organization; IQR, interquartile range; MIBC, muscle‐invasive bladder cancer; *n*, number; PE, pulmonary embolism; PIC, postoperative intravesical chemotherapy; PPO, Preferred Provider Organization; RC, radical cystectomy; RNU, radical nephroureterectomy; SD, standard deviation; SVT, superficial thrombophlebitis/phlebitis; TURBT, transurethral resection of bladder tumour; US, United States; UTUC, upper tract urothelial carcinoma; VTE, venous thromboembolism.

### 3.2 Outcomes

#### 3.2.1 VTE history and intra‐ and 90‐day postoperative sequelae

The effect of VTE events on intra‐ and 90‐day postoperative complications have been summarized and visually shown in Table 2.

**TABLE 2:** Multivariable logistic regression estimates for the risk of prespecified outcomes.

Outcomes	Preop‐VTE	No preop‐VTE	*p* Value	aOR (95% CI)	*p* Value
Complications, *n* (%)					
Any	343 (4.6)	4526 (3.4)	<0.0001	1.28 (1.14–1.43)	<0.0001
Haemorrhagic	129 (1.8)	1550 (1.2)	<0.0001	1.42 (1.18–1.71)	0.0002
Urinary tract related	75 (1.0)	1189 (0.9)	0.2930	1.09 (0.86–1.38)	0.4893
Infectious	54 (0.73)	501 (0.38)	<0.0001	1.69 (1.27–2.26)	0.0003
Digestive	21 (0.28)	441 (0.33)	0.4820	0.84 (0.54–1.30)	0.4316
Intraoperative	18 (0.2)	356 (0.3)	0.6886	0.96 (0.59–1.55)	0.8648
Cardiac	12 (0.2)	150 (0.1)	0.2245	1.18 (0.65–2.15)	0.5797
Respiratory	13 (0.18)	185 (0.14)	0.4164	1.07 (0.61–1.89)	0.8173
Length of stay, median days (IQR)	6 (3–11)	3 (1–6)	<0.0001	2.23 (1.90–2.62)	<0.0001
1st quartile	98 (12.2)	2220 (25.6)	<0.0001		
2nd quartile	148 (18.4)	2604 (30.0)			
3rd quartile	174 (21.6)	1882 (21.7)		2.51 (2.15–2.92)	<0.0001
4th quartile	384 (47.8)	1966 (22.7)			
Re‐hospitalization, 90 days	1540 (20.9)	17 614 (13.3)	<0.0001	1.47 (1.39–1.56)	<0.0001
Discharge status, *n* (%)			0.6185	0.96 (0.82–1.11)	0.5705
Home/self‐care	377 (46.9)	3987 (46.0)			
Other facilities	427 (53.1)	4685 (54.0)			
Median costs, median $ (IQR)	6990 (3316–19 760)	6498 (2891–15 015)	<0.0001	1.17 (1.12–1.23)	<0.0001
1st quartile	1708 (23.2)	33 239 (25.1)	<0.0001		
2nd quartile	1777 (24.1)	33 171 (25.1)			
3rd quartile	1691 (22.9)	33 257 (25.1)		1.24 (1.17–1.31)	<0.0001
4th quartile	2199 (29.8)	32 749 (24.7)			

Abbreviations: aOR, adjusted odds ratio; CI, confidence interval; IQR, interquartile range; *n*, number; VTE, venous thromboembolism.

The two most prevalent adverse events were haemorrhagic (1.8% (*n* = 129) preop‐VTE vs. 1.2% (*n* = 1550) no preop‐VTE) and urinary tract related (1.0% (*n* = 75) preop‐VTE vs. 0.9% (*n* = 1189) no preop‐VTE). Multivariable logistic regression analysis showed that preop‐VTE did not enhance risk of urinary tract related (adjusted odds ratio [aOR] 1.09, 95% CI 0.86–1.38), digestive (aOR 0.84, 95% CI 0.54–1.30; 0.28% (*n* = 21) preop‐VTE vs. 0.33% (*n* = 441) no preop‐VTE), intraoperative (aOR 0.96, 95% CI 0.59–1.55; 0.2% (*n* = 18) preop‐VTE vs. 0.3% (*n* = 356) no preop‐VTE), cardiac (aOR 1.18, 95% CI 0.65–2.15; 0.2% (*n* = 12) preop‐VTE vs. 0.1% (*n* = 150) no preop‐VTE) and respiratory (aOR 1.07, 95% CI 0.61–1.89; 0.18% (*n* = 13) preop‐VTE vs. 0.14% (*n* = 185) no preop‐VTE) complications. On the other hand, patients with a history of preoperative VTE events had significantly higher risk of developing any (aOR 1.28, 95% CI 1.14–1.43; 4.6% (*n* = 343) preop‐VTE vs. 3.4% (*n* = 4526) no preop‐VTE), infectious (aOR 1.69, 95% CI 1.27–2.26; 0.73% (*n* = 54) preop‐VTE vs. 0.38% (*n* = 501) no preop‐VTE) and haemorrhagic (aOR 1.42, 95% CI 1.18–1.71) postoperative complications.

In addition, sensitivity analysis based on VTE severity was performed. It was found that the history of DVT was not associated with a significantly higher probability of any, infectious, or haemorrhagic adverse events. However, preoperative PE and SVT significantly increased the risk of these complications (Table S2). Finally, only VTE before minor TURBT enhanced the risk of any, infectious and haemorrhagic complications (Table S3). It is worth mentioning that these associations were not significant for VTE before major TURBT.

In addition, significantly more patients from the preop‐VTE group (21.95%, *n* = 1619) developed postoperative VTE events, when compared with the no preop‐VTE group (1.48%, *n* = 1959). These findings were corroborated in multivariable analysis, which showed that a preoperative diagnosis of VTE is the strongest predictor for occurrence of novel VTE events following TURBT. This finding was consistent across all analyses, either in the case of any type of VTE (aOR 17.30, 95% CI 16.05–18.65) or separately with regards to specific VTE types (Table S2) or TURBT type (Table S3).

#### 3.2.2 VTE history and length of stay, discharge status and re‐hospitalization

The majority of TURBT procedures in this study were performed in the outpatient setting. Nevertheless, patients with a history of preoperative VTE events had a higher median length of stay compared to those without a diagnosis of VTE prior to TURBT (median 6 [IQR 3–11] vs. median 3 [IQR 1–6], respectively) (Table 2). The results of the multivariable logistic regression analysis confirmed an increased risk for prolonged hospitalization for preoperative VTE events both with the median (aOR 2.23, 95% CI 1.90–2.62) and the 3rd quartile distribution threshold (aOR 2.51, 95% CI 2.15–2.92). The results remained consistent after sub‐stratification for the degree of VTE severity (Table S2) and TURBT extent (Table S3).

A comparable proportion of patients in the preop‐VTE group (53.1%) were discharged to other medical facilities (e.g. hospice medical facilities, federal facilities, short‐term hospitals) rather than home or self‐care, compared to the no preop‐VTE group (54.0%) (Table 2). In multivariable analysis, a history of VTE prior to TURBT was not significantly associated with an increased probability of being discharged elsewhere rather than home or self‐care in case of any VTE events (aOR 0.96, 95% CI 0.82–1.11) and was largely irrespective of the VTE severity (Table S2) and TURBT type (Table S3).

The rates of re‐hospitalization within 90 days following TURBT were reported to be higher in patients with a preoperative VTE diagnosis than in individuals without a history of VTE before TURBT (20.9% vs. 13.3%). Multivariable analysis demonstrated that patients with VTE prior to TURBT had a higher probability of experiencing re‐hospitalization in case of any VTE events (aOR 1.47, 95% CI 1.39–1.56). The results remained unchanged after sub‐stratification for the degree of VTE severity (Table S2) and TURBT extent (Table S3).

#### 3.2.3 VTE history and total hospital costs

The analysis revealed that the calculated median healthcare cost for patients with recorded VTE prior to TURBT was $6990 (IQR 3316–$19 760), which was slightly increased (by approximately $500) compared to patients with no history of preoperative VTE (median $6498; IQR: $2891–$15 015). History of VTE events prior to TURBT was found to be a predictive factor associated with an increased probability of higher costs than either the median (aOR 1.17, 95% CI 1.12–1.23) or 3rd quartile distribution for total hospital expenditures (aOR 1.24, 95% CI 1.17–1.31) (Table 2). The results after sub‐stratification for the degree of VTE severity and TURBT extent were presented in Table S2 and Table S3, respectively.

## 4 DISCUSSION

This is the first study to our knowledge that has evaluated preoperative VTE as a risk factor associated with TURBT outcomes. Our retrospective cohort analysis revealed that patients undergoing TURBT with a known history of VTE events had increased morbidity, postoperative VTE events, prolonged hospital length of stay, higher readmission rates and increased hospital costs. We hope that this will be an important first step to build on this important body of literature to help improve patient care.

As previously mentioned, there has been limited data on endoscopic urologic procedures and postoperative VTE. However, a 2021 retrospective study by Zheng et al. assessed the risk of VTE following transurethral resection of prostate (TURP) in 451 patients and found that 8% of patients had postoperative VTE with history of preoperative VTE being the strongest preoperative independent risk factor (aOR 8.597, 95% CI 1.468–50.348).^20^, ^21^ Similarly in our study, there was a significantly increased odds of having postoperative VTE (aOR 17.30, 95% CI 16.05–18.65) in patients with preoperative VTE undergoing TURBT.

There has also been no published data to our knowledge that has assessed the preoperative risk of a VTE history in patients undergoing TURBT. We found in our study that patients with preoperative VTE undergoing TURBT had a significantly increased risk of not only haemorrhagic, infectious, but also any complication. The only urological study which has assessed preoperative VTE as an independent risk factor for postoperative complications is Patel et al.'s 2022 study, which similarly used a large healthcare dataset to assess the risk of preoperative VTE in patients undergoing radical versus partial nephrectomy. They found that these patients similarly had higher rates of minor and major complications, mortality and ICU admissions.^22^

Although TURBT is typically considered an outpatient surgery, it certainly carries a higher rate of readmissions compared to other outpatient surgeries. One prospective study of 17 638 patients undergoing outpatient surgery demonstrated a total readmission rate of 1.1% for all outpatient surgeries, but that of TURBT was 5.7%.^23^ A retrospective study reviewing the National Surgical Quality Improvement Program (NSQIP) database identified 153 228 outpatient procedures and found TURBT to have the highest number of overall readmissions at 4.97%.^24^ Predictors of readmission in patients undergoing TURBT include older age, higher ASA score, longer operative times, locally advanced or systemic BCa at baseline clinical (cT) assessment, bleeding disorders and male gender.^25^, ^26^, ^27^, ^28^, ^29^, ^30^ None of these studies specifically looked at preoperative VTE as a risk factor for readmission. When controlling for age, CCI >3, obesity and TURBT size, we found preoperative VTE increased the odds of readmission in all patients undergoing TURBT regardless of VTE subtype (aOR 1.47, 95% CI 1.39–1.56). Although typically an outpatient procedure, we additionally found that those patients who were admitted as inpatients had an increase in hospital length of stay. Given the increased odds for readmission and hospital length of stay, it is no surprise that these patients also have increased hospital associated costs.

Our study is not without limitations. This study utilizes insurance claims data and codes which could be inaccurate as well as lack of generalizability to the world over. Additionally, the study is retrospective in nature. Our database does not include information on pathologic information which may also influence our analysis. In addition, there is a possibility of underreporting of preoperative VTE given patients with asymptomatic preoperative VTE may have been missed. That being said, this paper is the first of its kind to our knowledge and this large dataset has allowed us to study this important topic with significant statistical power.

To conclude, TURBT is the typical initial treatment and diagnosis for patients with a bladder mass. Patients with a history of preoperative VTE undergoing TURBT are found to have increased: morbidity, postoperative VTE events, hospital length of stay, costs and readmission rates. This data will help us in counselling patients about the risks associated with surgery and add to the scarce body of literature on the influence of VTE in endoscopic urologic procedures.

## AUTHOR CONTRIBUTIONS

*Full access to all the data in the study and integrity of the data and the accuracy of the data analysis*: Francesco Del Giudice. *Study concept and design*: Anas S. Tresh, Francesco Del Giudice, Ettore De Berardinis and Benjamin I. Chung. *Acquisition of data*: Shufeng Li. *Analysis and interpretation of data*: Shufeng Li, Satvir Basran, Dalila Carino and Valerio Santarelli. *Drafting of the manuscript*: Anas S. Tresh, Francesco Del Giudice and Jan Łaszkiewicz. *Critical revision of the manuscript for important intellectual content*: Bernardo Rocco, Roman Mayr, Matteo Ferro, Riccardo Autorino, Felice Crocetto, Renate Pichler, Jose Daniel Subiela, Jorge Cano Velasco, Marco Moschini, Andrea Mari, Andrea Gallioli, Francesco Soria, Simone Albisinni and Wojciech Krajewski. *Statistical analysis*: Shufeng Li and Łukasz Nowak. *Supervision*: Ettore De Berardinis, Maria Chiara Shighinolfi, Gabriele Bignante, Biagio Barone, Tomasz Szydełko, Benjamin Challacombe, Rajesh Nair and Benjamin I. Chung.

## CONFLICT OF INTEREST STATEMENT

Authors have no conflict of interest to disclose.

## RELEVANT DISCLOSURES

Authors have nothing to disclose.

## Supporting information


**Figure S1.** Flow chart design of the study summarizing analytic steps to achieve the final cohort of n = 139 800 patients with bladder cancer diagnosis undergoing transurethral resection of bladder tumour according to prespecified inclusion/exclusion criteria. **TURBT:** transurethral resection of bladder tumour; **n**: number; **BCa**: bladder cancer.

**Table S1.** List of ICD‐9/10, CPT, and HCPCS codes for bladder cancer diagnoses, venous thromboembolism events, surgical management (i.e., transurethral resection of bladder tumour) and other affiliated diagnoses and procedures.

**Table S2.** Multivariable adjusted logistic regression estimates for the risk of 90‐days postoperative complications and other prespecified outcomes according to the history of severity of venous thromboembolism events. **VTE**: venous thromboembolism**; aOR**: adjusted Odds Ratio; **CI**: confidence interval; **n**: number; **PE**: pulmonary embolism; **DVT**: deep venous thrombosis; **SVT**: superficial phlebitis/thrombophlebitis.

**Table S3.** Multivariable adjusted logistic regression estimates for the risk of 90‐days postoperative complications and other prespecified outcomes according to minor vs major TURBT. **TURBT**: transurethral resection of bladder tumour; **VTE**: venous thromboembolism**; aOR**: adjusted Odds Ratio; **CI**: confidence interval; **n**: number
