# Supplementary figures and images for: The impact of preoperative venous thromboembolism on patients undergoing TURBT: Perioperative outcomes and healthcare costs from US insurance claims data
Source: BJUI Compass. 2025 Jan 14;6(1):e481. doi: 10.1002/bco2.481 (PMC11771507; doi:10.1002/bco2.481)

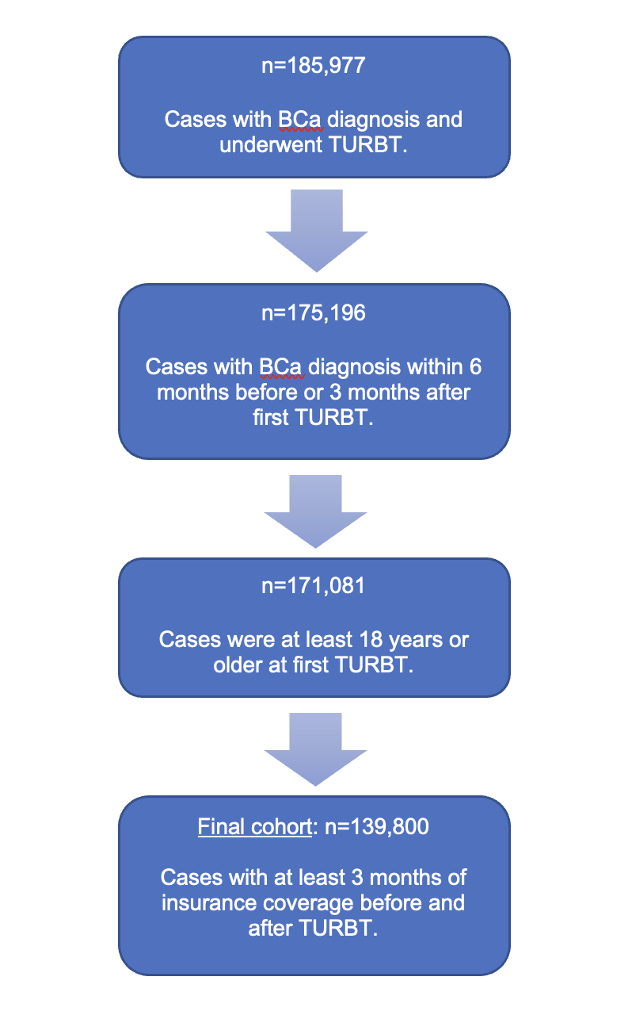

Supplement: Supplementary file 1 — Figure S1. Flow chart design of the study summarizing analytic steps to achieve the final cohort of n = 139 800 patients with bladder cancer diagnosis undergoing transurethral resection of bladder tumour according to prespecified inclusion/exclusion criteria. TURBT: transurethral resection of bladder tumour; n: number; BCa: bladder cancer. [file BCO2-6-e481-s004.png]
