# Supplementary material for: The impact of preoperative venous thromboembolism on patients undergoing TURBT: Perioperative outcomes and healthcare costs from US insurance claims data
Source: BJUI Compass. 2025 Jan 14;6(1):e481. doi: 10.1002/bco2.481 (PMC11771507; doi:10.1002/bco2.481)
Supplement: Supplementary file 2 — Table S1. List of ICD‐9/10, CPT, and HCPCS codes for bladder cancer diagnoses, venous thromboembolism events, surgical management (i.e., transurethral resection of bladder tumour) and other affiliated diagnoses and procedures. [file BCO2-6-e481-s002.docx]

| **Bladder Cancer codes by ICD9 and ICD10** | | | | | | |
| --- | --- | --- | --- | --- | --- | --- |
| **ICD-9** | **Description** | | | | | |
| 188.0 | Malignant neoplasm of bladder | | | | | |
| 188.1 | Malignant neoplasm of dome of bladder | | | | | |
| 188.2 | Malignant neoplasm of lateral wall of bladder | | | | | |
| 188.3 | Malignant neoplasm of anterior wall of bladder | | | | | |
| 188.4 | Malignant neoplasm of posterior wall of bladder | | | | | |
| 188.5 | Malignant neoplasm of bladder neck | | | | | |
| 188.6 | Malignant neoplasm of ureteric orifice | | | | | |
| 188.7 | Malignant neoplasm of urachus | | | | | |
| 188.8 | Malignant neoplasm of overlapping sites of bladder | | | | | |
| 188.9 | Malignant neoplasm of bladder, unspecified | | | | | |
| 189.3 | Malignant neoplasm of urethra | | | | | |
| 189.4 | Malignant neoplasm of paraurethral glands | | | | | |
| 198.1 | Secondary malignant neoplasm of other urinary organs | | | | | |
| 223.3 | Benign neoplasm of bladder | | | | | |
| 223.81 | Benign neoplasm of urethra | | | | | |
| 233.7 | Carcinoma in situ of bladder | | | | | |
| 236.7 | Neoplasm of uncertain behavior of bladder | | | | | |
| 236.90 | Neoplasm of uncertain behavior of urinary organ, unspecified | | | | | |
| **ICD-10** | **Description** | | | | | |
| C67.0 | Malignant neoplasm of trigone of bladder | | | | | |
| C67.1 | Malignant neoplasm of dome of bladder | | | | | |
| C67.2 | Malignant neoplasm of lateral wall of bladder | | | | | |
| C67.3 | Malignant neoplasm of anterior wall of bladder | | | | | |
| C67.4 | Malignant neoplasm of posterior wall of bladder | | | | | |
| C67.5 | Malignant neoplasm of bladder neck | | | | | |
| C67.6 | Malignant neoplasm of ureteric orifice | | | | | |
| C67.7 | Malignant neoplasm of urachus | | | | | |
| C67.8 | Malignant neoplasm of overlapping sites of bladder | | | | | |
| C67.9 | Malignant neoplasm of bladder, unspecified | | | | | |
| C68.0 | Malignant neoplasm of urethra | | | | | |
| C68.1 | Malignant neoplasm of paraurethral glands | | | | | |
| D09.0 | Carcinoma in situ of bladder | | | | | |
| D09.10 | Carcinoma in situ of unspecified urinary organ | | | | | |
| D09.19 | Carcinoma in situ of other urinary organs | | | | | |
| D09.9 | Carcinoma in situ, unspecified | | | | | |
| **TURBT codes by ICD9-CM, ICD10-PCS, and CPT** | | | | | | |
| **ICD-9-CM** | | | | | **Description** | |
| 57.49 | | | | | Other transurethral excision or destruction of lesion or tissue of bladder | |
| 57.39 | | | | | Other transurethral excision or destruction of lesion or tissue of bladder | |
| 57.33 | | | | | Closed [transurethral] biopsy of bladder | |
| **ICD-10-PCS** | | | | | **Description** | |
| 0T5B7ZZ | | | | | Destruction of Bladder, Via Natural or Artificial Opening | |
| 0T5B8ZZ | | | | | Destruction of Bladder, Via Natural or Artificial Opening Endoscopic | |
| 0T5C7ZZ | | | | | Destruction of Bladder Neck, Via Natural or Artificial Opening | |
| 0T5C8ZZ | | | | | Destruction of Bladder Neck, Via Natural or Artificial Opening Endoscopic | |
| 0TBB7ZZ | | | | | Excision of Bladder, Via Natural or Artificial Opening | |
| 0TBB8ZZ | | | | | Excision of Bladder, Via Natural or Artificial Opening Endoscopic | |
| 0TBC7ZZ | | | | | Excision of Bladder Neck, Via Natural or Artificial Opening | |
| 0TBC8ZZ | | | | | Excision of Bladder Neck, Via Natural or Artificial Opening Endoscopic | |
| 0TJB4ZZ | | | | | Inspection of Bladder, Percutaneous Endoscopic Approach | |
| 0TJB8ZZ | | | | | Inspection of Bladder, Via Natural or Artificial Opening Endoscopic | |
| 0TBB3ZX | | | | | Excision of Bladder, Percutaneous Approach, Diagnostic | |
| 0TBB4ZX | | | | | Excision of Bladder, Percutaneous Endoscopic Approach, Diagnostic | |
| 0TBB7ZX | | | | | Excision of Bladder, Via Natural or Artificial Opening, Diagnostic | |
| 0TBB8ZX | | | | | Excision of Bladder, Via Natural or Artificial Opening Endoscopic, Diagnostic | |
| 0TCB7ZZ | | | | | Extirpation of Matter from Bladder, Via Natural or Artificial Opening | |
| 0TCB8ZZ | | | | | Extirpation of Matter from Bladder, Via Natural or Artificial Opening Endoscopic | |
| 0TNB7ZZ | | | | | Release Bladder, Via Natural or Artificial Opening | |
| 0TNB8ZZ | | | | | Release Bladder, Via Natural or Artificial Opening Endoscopic | |
| 0TNC8ZZ | | | | | Release Bladder Neck, Via Natural or Artificial Opening Endoscopic | |
| 0TBC8ZX | | | | | Excision of Bladder Neck, Via Natural or Artificial Opening Endoscopic | |
| **CPT** | | | | | **Description** | |
| 52234 | | | | | Cystourethroscopy with fulguration (including cryosurgery or laser surgery) and or resection of  SMALL bladder tumor(s) (0.5 up to 2.0 cm) | |
| 52235 | | | | | Cystourethroscopy with fulguration (including cryosurgery or laser surgery) and or resection of  MEDIUM bladder tumor(s) (2.0 to 5.0 cm) | |
| 52240 | | | | | Cystourethroscopy with fulguration (including cryosurgery or laser surgery) and or resection of  LARGE bladder tumor(s) (>5.0 cm) | |
| 52224 | | | | | Trans-uretrhal fulguration of bladder tumor | |
| 51530 | | | | | Trans-uretrhal resection of bladder tumor | |
| 51020 | | | | | fulguration or cryosurgical destruction of bladder lesions through an incision | |
| 5130 | | | | | fulguration or cryosurgical destruction of bladder lesions through an incision | |
| 52204 | | | | | Cystourethroscopy, with biopsy(s) | |
| 52300 | | | | | Urethra and Bladder Transurethral Surgical Procedures. | |
| 52301 | | | | | Urethra and Bladder Transurethral Surgical Procedures. | |
| 52305 | | | | | Urethra and Bladder Transurethral Surgical Procedures. | |
| 52005 | | | | | Endoscopy-Cystoscopy, Urethroscopy, Cystourethroscopy Procedures on the Bladder. | |
| 52007 | | | | | Endoscopy-Cystoscopy, Urethroscopy, Cystourethroscopy Procedures on the Bladder. | |
| 52214 | | | | | Urethra and Bladder Transurethral Surgical Procedures. | |
| **Venous Thromboembolism by ICD9 and ICD10 codes** | | | | | | |
| **ICD-9** | | | | | | **Description** |
| 415.1 | | | | | | Pulmonary embolism and infarction |
| 415.13 | | | | | | Saddle embolus of pulmonary artery convert |
| 415.19 | | | | | | Other pulmonary embolism and infarction convert |
| 451.11 | | | | | | Phlebitis and thrombophlebitis of femoral vein (deep) (superficial) |
| 451.19 | | | | | | Phlebitis and thrombophlebitis of deep veins of lower extremities, other |
| 451.2 | | | | | | Phlebitis and thrombophlebitis of lower extremities, unspecified |
| 451.81 | | | | | | Phlebitis and thrombophlebitis of iliac vein |
| 452 | | | | | | Portal vein thrombosis |
| 453.3 | | | | | | Other venous embolism and thrombosis of renal vein |
| 453.40 | | | | | | Acute venous embolism and thrombosis of unspecified deep vessels of lower extremity |
| 453.41 | | | | | | Acute venous embolism and thrombosis of deep vessels of proximal lower extremity |
| 453.42 | | | | | | Acute venous embolism and thrombosis of deep vessels of distal lower extremity |
| 453.6 | | | | | | Venous embolism and thrombosis of superficial vessels of lower extremity |
| 453.8 | | | | | | Acute venous embolism and thrombosis of other specified veins |
| 453.81 | | | | | | Acute venous embolism and thrombosis of superficial veins of upper extremity |
| 453.82 | | | | | | Acute venous embolism and thrombosis of deep veins of upper extremity |
| 453.83 | | | | | | Acute venous embolism and thrombosis of upper extremity, unspecified |
| **ICD-10** | | | | | | **Description** |
| I26.92 | | | | | | - Saddle embolus of pulmonary artery without acute cor pulmonale |
| I26.99 | | | | | | Other pulmonary embolism without acute cor pulmonale |
| I80.0 | | | | | | Phlebitis and thrombophlebitis of superficial vessels of lower extremities |
| I80.00 | | | | | | Phlebitis and thrombophlebitis of superficial vessels of unspecified lower extremity |
| I80.01 | | | | | | Phlebitis and thrombophlebitis of superficial vessels of right lower extremity |
| I80.02 | | | | | | Phlebitis and thrombophlebitis of superficial vessels of left lower extremity |
| I80.03 | | | | | | …… bilateral |
| I80.1 | | | | | | Phlebitis and thrombophlebitis of femoral vein |
| I80.10 | | | | | | Phlebitis and thrombophlebitis of unspecified femoral vein |
| I80.11 | | | | | | Phlebitis and thrombophlebitis of right femoral vein |
| I80.12 | | | | | | Phlebitis and thrombophlebitis of left femoral vein |
| I80.13 | | | | | | …… bilateral |
| I80.2 | | | | | | Phlebitis and thrombophlebitis of other and unspecified deep vessels of lower extremities |
| I80.20 | | | | | | Phlebitis and thrombophlebitis of unspecified deep vessels of lower extremities |
| I80.201 | | | | | | Phlebitis and thrombophlebitis of unspecified deep vessels of right lower extremity |
| I80.202 | | | | | | Phlebitis and thrombophlebitis of unspecified deep vessels of left lower extremity |
| I80.203 | | | | | | …… bilateral |
| I80.209 | | | | | | Phlebitis and thrombophlebitis of unspecified deep vessels of unspecified lower extremity |
| I80.21 | | | | | | Phlebitis and thrombophlebitis of iliac vein |
| I80.211 | | | | | | Phlebitis and thrombophlebitis of right iliac vein |
| I80.212 | | | | | | Phlebitis and thrombophlebitis of left iliac vein |
| I80.213 | | | | | | …… bilateral |
| I80.219 | | | | | | Phlebitis and thrombophlebitis of unspecified iliac vein |
| I80.22 | | | | | | Phlebitis and thrombophlebitis of popliteal vein |
| I80.221 | | | | | | Phlebitis and thrombophlebitis of right popliteal vein |
| I80.222 | | | | | | Phlebitis and thrombophlebitis of left popliteal vein |
| I80.223 | | | | | | …… bilateral |
| I80.229 | | | | | | Phlebitis and thrombophlebitis of unspecified popliteal vein |
| I80.23 | | | | | | Phlebitis and thrombophlebitis of tibial vein |
| I80.231 | | | | | | Phlebitis and thrombophlebitis of right tibial vein |
| I80.232 | | | | | | Phlebitis and thrombophlebitis of left tibial vein |
| I80.233 | | | | | | …… bilateral |
| I80.239 | | | | | | Phlebitis and thrombophlebitis of unspecified tibial vein |
| I80.24 | | | | | | Phlebitis and thrombophlebitis of peroneal vein |
| I80.241 | | | | | | Phlebitis and thrombophlebitis of right peroneal vein |
| I80.242 | | | | | | Phlebitis and thrombophlebitis of left peroneal vein |
| I80.243 | | | | | | …… bilateral |
| I80.249 | | | | | | Phlebitis and thrombophlebitis of unspecified peroneal vein |
| I80.25 | | | | | | Phlebitis and thrombophlebitis of calf muscular vein |
| I80.251 | | | | | | Phlebitis and thrombophlebitis of right calf muscular vein |
| I80.252 | | | | | | Phlebitis and thrombophlebitis of left calf muscular vein |
| I80.253 | | | | | | …… bilateral |
| I80.259 | | | | | | Phlebitis and thrombophlebitis of unspecified calf muscular vein |
| I80.29 | | | | | | Phlebitis and thrombophlebitis of other deep vessels of lower extremities |
| I80.291 | | | | | | Phlebitis and thrombophlebitis of other deep vessels of right lower extremity |
| I80.292 | | | | | | Phlebitis and thrombophlebitis of other deep vessels of left lower extremity |
| I80.293 | | | | | | Phlebitis and thrombophlebitis of other deep vessels of lower extremity, bilateral |
| I80.299 | | | | | | Phlebitis and thrombophlebitis of other deep vessels of unspecified lower extremity |
| I80.3 | | | | | | Phlebitis and thrombophlebitis of lower extremities, unspecified |
| I80.8 | | | | | | Phlebitis and thrombophlebitis of other sites |
| I80.9 | | | | | | Phlebitis and thrombophlebitis of unspecified site |
| I81 | | | | | | Portal vein thrombosis |
| I82 | | | | | | Other venous embolism and thrombosis |
| I82.2 | | | | | | Embolism and thrombosis of vena cava and other thoracic veins |
| I82.21 | | | | | | Embolism and thrombosis of superior vena cava |
| I82.210 | | | | | | Acute embolism and thrombosis of superior vena cava |
| I82.22 | | | | | | Embolism and thrombosis of inferior vena cava |
| I82.220 | | | | | | Acute embolism and thrombosis of inferior vena cava |
| I82.29 | | | | | | Embolism and thrombosis of other thoracic veins |
| I82.290 | | | | | | Acute embolism and thrombosis of other thoracic veins |
| I82.6 | | | | | | Acute embolism and thrombosis of veins of upper extremity |
| I82.60 | | | | | | Acute embolism and thrombosis of unspecified veins of upper extremity |
| I82.601 | | | | | | Acute embolism and thrombosis of unspecified veins of right upper extremity |
| I82.602 | | | | | | Acute embolism and thrombosis of unspecified veins of left upper extremity |
| I82.603 | | | | | | …… bilateral |
| I82.609 | | | | | | Acute embolism and thrombosis of unspecified veins of unspecified upper extremity |
| I82.61 | | | | | | Acute embolism and thrombosis of superficial veins of upper extremity |
| I82.611 | | | | | | Acute embolism and thrombosis of superficial veins of right upper extremity |
| I82.612 | | | | | | Acute embolism and thrombosis of superficial veins of left upper extremity |
| I82.613 | | | | | | …… bilateral |
| I82.619 | | | | | | Acute embolism and thrombosis of superficial veins of unspecified upper extremity |
| I82.62 | | | | | | Acute embolism and thrombosis of deep veins of upper extremity |
| I82.621 | | | | | | Acute embolism and thrombosis of deep veins of right upper extremity |
| I82.622 | | | | | | Acute embolism and thrombosis of deep veins of left upper extremity |
| I82.623 | | | | | | …… bilateral |
| I82.629 | | | | | | Acute embolism and thrombosis of deep veins of unspecified upper extremity |
| I82.8 | | | | | | Embolism and thrombosis of other specified veins |
| I82.A | | | | | | Embolism and thrombosis of axillary vein |
| I82.A1 | | | | | | Acute embolism and thrombosis of axillary vein |
| I82.A11 | | | | | | Acute embolism and thrombosis of right axillary vein |
| I82.A12 | | | | | | Acute embolism and thrombosis of left axillary vein |
| I82.A13 | | | | | | …… bilateral |
| I82.A19 | | | | | | Acute embolism and thrombosis of unspecified axillary vein |
| I82.B | | | | | | Embolism and thrombosis of subclavian vein |
| I82.B1 | | | | | | Acute embolism and thrombosis of subclavian vein |
| I82.B11 | | | | | | Acute embolism and thrombosis of right subclavian vein |
| I82.B12 | | | | | | Acute embolism and thrombosis of left subclavian vein |
| I82.B13 | | | | | | …… bilateral |
| I82.B19 | | | | | | Acute embolism and thrombosis of unspecified subclavian vein |
| I82.C | | | | | | Embolism and thrombosis of internal jugular vein |
| I82.C1 | | | | | | Acute embolism and thrombosis of internal jugular vein |
| I82.C11 | | | | | | Acute embolism and thrombosis of right internal jugular vein |
| I82.C12 | | | | | | Acute embolism and thrombosis of left internal jugular vein |
| I82.C13 | | | | | | …… bilateral |
| I82.C19 | | | | | | Acute embolism and thrombosis of unspecified internal jugular vein |
| **Intravesical chemo/immunotherapy codes by CPT and HCPCS** | | | | | | |
| **CPT** | | | | **Description** | | |
| 51720 | | | | Bladder instillation of anticarcinogenic agent | | |
| 90586 | | | | Bacillus Calmette-Guérin vaccine for bladder cancer, live, for intravesical use | | |
| 96401 | | | | Chemotherapy administration, intravesical or bladder via catheter | | |
| **HCPCS** | | | | **Description** | | |
| J9030 | | | | BCG Live Intravesical Instillation after July 2019 | | |
| J9031 | | | | BCG Live Intravesical Instillation before July 2019 | | |
| **Radical cystectomy codes by ICD9-CM, ICD10-PCS, and CPT** | | | | | | |
| **ICD-9-CM** | | | **Description** | | | |
| 57.6 | | | Partial Cystectomy | | | |
| 57.7 | | | Total Cystectomy | | | |
| 57.71 | | | Radical Cystectomy | | | |
| 57.79 | | | Other Total Cystectomy | | | |
| **ICD-10-PCS** | | | **Description** | | | |
| 0TBB0ZZ | | | Excision of Bladder, Open Approach | | | |
| 0TBB3ZZ | | | Excision of Bladder, Percutaneous Approach | | | |
| 0TBB4ZZ | | | Resection of Bladder, Percutaneous Endoscopic Approach | | | |
| 0TBB7ZZ | | | Resection of Bladder, Via Natural or Artificial Opening | | | |
| 0TBB8ZZ | | | Resection of Bladder, Via Natural or Artificial Opening Endoscopic | | | |
| **CPT** | | | **Description** | | | |
| 51550 | | | Cystectomy, partial | | | |
| 51555 | | | Cystectomy, partial, complicated | | | |
| 51565 | | | Cystectomy, partial, with ureteral reimplantation | | | |
| 51570 | | | Cystectomy, complete (separate procedure) | | | |
| 51575 | | | Cystectomy, with bilateral pelvic lymphadenectomy | | | |
| 51580 | | | Cystectomy, complete, with ureterosigmoidostomy or ureterocutaneous diversion | | | |
| 51585 | | | Cystectomy, with ureterosigmoidostomy or ureterocutaneous diversion, with bilateral pelvic lymphadenectomy | | | |
| 51590 | | | Cystectomy, with ileal conduit or sigmoid bladder | | | |
| 51595 | | | Cystectomy, with ileal conduit or sigmoid bladder, with bilateral pelvic lymphadenectomy | | | |
| 51596 | | | Cystectomy, with continent urinary diversion, and open technique | | | |
| 51597 | | | Pelvic exenteration | | | |
| **Upper tract urothelial carcinoma codes by ICD9 and ICD10** | | | | | | |
| **ICD-9** | | | **Description** | | | |
| 189.1 | | | Malignant neoplasm of renal pelvis | | | |
| 189.2 | | | Malignant neoplasm of ureter | | | |
| 189.8 | | | Malignant neoplasm of other specified sites of urinary organs | | | |
| 189.9 | | | Malignant neoplasm of urinary organ, site unspecified | | | |
| **ICD-10** | | | **Description** | | | |
| C65.1 | | | Malignant neoplasm of right renal pelvis | | | |
| C65.2 | | | Malignant neoplasm of left renal pelvis | | | |
| C65.9 | | | Malignant neoplasm of unspecified renal pelvis | | | |
| C66.1 | | | Malignant neoplasm of right ureter | | | |
| C66.2 | | | Malignant neoplasm of left ureter | | | |
| C66.9 | | | Malignant neoplasm of unspecified ureter | | | |
| C68.8 | | | Malignant neoplasm of overlapping sites of urinary organs | | | |
| C68.9 | | | Malignant neoplasm of urinary organ, unspecified | | | |
| **Nephroureterectomy codes by ICD9-CM, ICD10-PCS, and CPT** | | | | | | |
| **ICD-9-CM** | | | **Description** | | | |
| 56.4 | | | Ureterectomy | | | |
| 56.40 | | | Ureterectomy, not otherwise specified | | | |
| 56.41 | | | Partial ureterectomy | | | |
| 56.42 | | | Total ureterectomy | | | |
| 55.51 | | | Nephroureterectomy | | | |
| **ICD-10-PCS** | | | **Description** | | | |
| 0TB60ZZ | | | Excision of Right Ureter, Open Approach | | | |
| 0TB63ZZ | | | Excision of Right Ureter, Percutaneous Approach | | | |
| 0TB64ZZ | | | Excision of Right Ureter, Percutaneous Endoscopic Approach | | | |
| 0TB67ZZ | | | Excision of Right Ureter, Via Natural or Artificial Opening | | | |
| 0TB68ZZ | | | Excision of Right Ureter, Via Natural or Artificial Opening Endoscopic | | | |
| 0TB70ZZ | | | Excision of Left Ureter, Open Approach | | | |
| 0TB73ZZ | | | Excision of Left Ureter, Percutaneous Approach | | | |
| 0TB74ZZ | | | Excision of Left Ureter, Percutaneous Endoscopic Approach | | | |
| 0TB77ZZ | | | Excision of Left Ureter, Via Natural or Artificial Opening | | | |
| 0TB78ZZ | | | Excision of Left Ureter, Via Natural or Artificial Opening Endoscopic | | | |
| 0TT60ZZ | | | Resection of Right Ureter, Open Approach | | | |
| 0TT64ZZ | | | Resection of Right Ureter, Percutaneous Endoscopic Approach | | | |
| 0TT67ZZ | | | Resection of Right Ureter, Via Natural or Artificial Opening | | | |
| 0TT68ZZ | | | Resection of Right Ureter, Via Natural or Artificial Opening Endoscopic | | | |
| 0TT70ZZ | | | Resection of Left Ureter, Open Approach | | | |
| 0TT74ZZ | | | Resection of Left Ureter, Percutaneous Endoscopic Approach | | | |
| 0TT77ZZ | | | Resection of Left Ureter, Via Natural or Artificial Opening | | | |
| 0TT78ZZ | | | Resection of Left Ureter, Via Natural or Artificial Opening Endoscopic | | | |
| 0TT00ZZ | | | Resection of Right Kidney, Open Approach | | | |
| 0TT04ZZ | | | Resection of Right Kidney, Percutaneous Endoscopic Approach | | | |
| 0TT10ZZ | | | Resection of Left Kidney, Open Approach | | | |
| 0TT14ZZ | | | Resection of Left Kidney, Percutaneous Endoscopic Approach | | | |
| **CPT** | | | **Description** | | | |
| 50220 | | | Incision Procedures on the Kidney | | | |
| 50225 | | | Incision Procedures on the Kidney | | | |
| 50230 | | | Incision Procedures on the Kidney | | | |
| 50234 | | | Incision Procedures on the Kidney (with bladder cuff) | | | |
| 50236 | | | Incision Procedures on the Kidney (with bladder cuff), Open Approach | | | |
| 50650 | | | Excision Procedures on the Ureter (only ureterectomy) | | | |
| 50660 | | | Excision Procedures on the Ureter (only ureterectomy | | | |
| 50544 | | | Laparoscopic Procedures on the Kidney | | | |
| 50545 | | | Laparoscopic Procedures on the Kidney | | | |
| **Antibiotics outpatient therapeutic class by Market Scan dictionary** | | | | | | |
| **Code** | | **Description** | | | | |
| 4 | | aminoglycosides | | | | |
| 5 | | antifungals | | | | |
| 6 | | cephalosporins | | | | |
| 7 | | beta lactams | | | | |
| 9 | | macrolides | | | | |
| 10 | | penicillin’s | | | | |
| 11 | | tetracyclines | | | | |
| 12 | | miscellaneous | | | | |
| 16 | | quinolones | | | | |
| 17 | | sulfonamides | | | | |
| 18 | | sulfones | | | | |
| 19 | | Urinary anti-infectives | | | | |
| 20 | | Anti-infectives miscellaneous | | | | |
| 133 | | antiinfection, antibiotics | | | | |
| 135 | | sulfonamides | | | | |
| 136 | | anti-infective miscellaneous | | | | |
| 190 | | Anti-infectives, antibiotics | | | | |
| 192 | | Anti-infectives, antifungals | | | | |
| 194 | | Anti-infectives local miscellaneous | | | | |
| 290 | | antifungal | | | | |
| **Thromboprophylaxis outpatient class searched by Market Scan dictionary** | | | | | | |
| Dalteparin | | | | | | |
| Bemiparin | | | | | | |
| Enoxaparin | | | | | | |
| Nadroparin | | | | | | |
| Parnaparin | | | | | | |
| Fondaparinux | | | | | | |
| Warfarin | | | | | | |
| Phenprocoumon | | | | | | |
| Acenocumarol | | | | | | |
| Dabigatran | | | | | | |
| Apixaban | | | | | | |
| Edoxaban | | | | | | |
| Rivaroxaban | | | | | | |
