# Supplementary material for: The impact of preoperative venous thromboembolism on patients undergoing TURBT: Perioperative outcomes and healthcare costs from US insurance claims data
Source: BJUI Compass. 2025 Jan 14;6(1):e481. doi: 10.1002/bco2.481 (PMC11771507; doi:10.1002/bco2.481)
Supplement: Supplementary file 3 — Table S2. Multivariable adjusted logistic regression estimates for the risk of 90‐days postoperative complications and other prespecified outcomes according to the history of severity of venous thromboembolism events. VTE: venous thromboembolism; aOR: adjusted Odds Ratio; CI: confidence interval; n: number; PE: pulmonary embolism; DVT: deep venous thrombosis; SVT: superficial phlebitis/thrombophlebitis. [file BCO2-6-e481-s001.docx]

| **Complications** | **Type of preop-VTE event** | **aOR** (95% CI) | ***p-value*** |
| --- | --- | --- | --- |
| **Intraoperative** | PE vs. No preop-VTE | 1.80 (0.92 - 3.50) | 0.0849 |
|  | DVT vs. No preop-VTE | 0.76 (0.34 - 1.72) | 0.5147 |
|  | SVT vs. No preop-VTE | 0.51 (0.16 - 1.59) | 0.2448 |
| **Any** | PE vs. No preop-VTE | 1.49 (1.22 - 1.81) | <.0001 |
|  | DVT vs. No preop-VTE | 1.11 (0.93 - 1.33) | 0.2545 |
|  | SVT vs. No preop-VTE | 1.33 (1.09 - 1.62) | 0.0047 |
| **Respiratory** | PE vs. No preop-VTE | 1.23 (0.45 - 3.33) | 0.6845 |
|  | DVT vs. No preop-VTE | 0.79 (0.29 - 2.15) | 0.6498 |
|  | SVT vs. No preop-VTE | 1.29 (0.53 - 3.16) | 0.5718 |
| **Digestive** | PE vs. No preop-VTE | 0.87 (0.39 - 1.96) | 0.7387 |
|  | DVT vs. No preop-VTE | 0.57 (0.25 - 1.27) | 0.1671 |
|  | SVT vs. No preop-VTE | 1.19 (0.61 - 2.31) | 0.6145 |
| **Infectious** | PE vs. No preop-VTE | 1.80 (1.09 - 2.98) | 0.0220 |
|  | DVT vs. No preop-VTE | 1.40 (0.88 - 2.22) | 0.1561 |
|  | SVT vs. No preop-VTE | 2.01 (1.26 - 3.20) | 0.0032 |
| **Hemorrhagic** | PE vs. No preop-VTE | 1.67 (1.22 - 2.28) | 0.0015 |
|  | DVT vs. No preop-VTE | 1.10 (0.81 - 1.50) | 0.5477 |
|  | SVT vs. No preop-VTE | 1.64 (1.22 - 2.22) | 0.0011 |
| **Urinary tract related** | PE vs. No preop-VTE | 1.45 (0.98 - 2.13) | 0.0607 |
|  | DVT vs. No preop-VTE | 1.08 (0.75 - 1.54) | 0.6960 |
|  | SVT vs. No preop-VTE | 0.79 (0.49 - 1.28) | 0.3443 |
| **Cardiac** | PE vs. No preop-VTE | 1.08 (0.34 - 3.42) | 0.8900 |
|  | DVT vs. No preop-VTE | 1.18 (0.48 - 2.90) | 0.7148 |
|  | SVT vs. No preop-VTE | 1.27 (0.47 - 3.46) | 0.6355 |
| **Postoperative VTE**, new events | PE vs. No preop-VTE | 31.98 (28.78 - 35.54) | <.0001 |
|  | DVT vs. No preop-VTE | 18.33 (16.62 - 20.22) | <.0001 |
|  | SVT vs. No preop-VTE | 6.44 (5.54 - 7.48) | <.0001 |
| **Outcomes** | **Type of preop-VTE event** | **aOR** (95% CI) | ***p-value*** |
| **Median costs**, median $ | PE vs. No preop-VTE | 1.27 (1.16 - 1.39) | <.0001 |
|  | DVT vs. No preop-VTE | 1.25 (1.16 - 1.34) | <.0001 |
|  | SVT vs. No preop-VTE | 1.00 (0.92 - 1.10) | 0.9351 |
| **Re-hospitalization**, within 90-days | PE vs. No preop-VTE | 1.62 (1.46 - 1.81) | <.0001 |
|  | DVT vs. No preop-VTE | 1.52 (1.39 - 1.66) | <.0001 |
|  | SVT vs. No preop-VTE | 1.28 (1.15 - 1.43) | <.0001 |
| **Length of stay**, median days | PE vs. No preop-VTE | 2.44 (1.80 - 3.31) | <.0001 |
|  | DVT vs. No preop-VTE | 2.80 (2.19 - 3.59) | <.0001 |
|  | SVT vs. No preop-VTE | 1.50 (1.14 - 1.98) | 0,0043 |
| **Discharge status**, other than home | PE vs. No preop-VTE | 1.06 (0.80 - 1.41) | 0.6714 |
|  | DVT vs. No preop-VTE | 1.11 (0.89 - 1.38) | 0.3715 |
|  | SVT vs. No preop-VTE | 0.69 (0.53 - 0.91) | 0.0084 |
