# Supplementary material for: The impact of preoperative venous thromboembolism on patients undergoing TURBT: Perioperative outcomes and healthcare costs from US insurance claims data
Source: BJUI Compass. 2025 Jan 14;6(1):e481. doi: 10.1002/bco2.481 (PMC11771507; doi:10.1002/bco2.481)
Supplement: Supplementary file 4 — Table S3. Multivariable adjusted logistic regression estimates for the risk of 90‐days postoperative complications and other prespecified outcomes according to minor vs major TURBT. TURBT: transurethral resection of bladder tumour; VTE: venous thromboembolism; aOR: adjusted Odds Ratio; CI: confidence interval; n: number [file BCO2-6-e481-s003.docx]

| **Complications** | **Extent of TURBT (preop-VTE vs. no preop-VTE)** | **aOR** (95%CI) | ***p-value*** |
| --- | --- | --- | --- |
| **Intraoperative** | Minor | 1.12 (0.62 - 2.03) | 0.7008 |
|  | Major | 0.74 (0.32 - 1.68) | 0.4653 |
| **Any** | Minor | 1.42 (1.23 - 1.63) | <.0001 |
|  | Major | 1.07 (0.88 - 1.30) | 0.4833 |
| **Respiratory** | Minor | 1.21 (0.58 - 2.52) | 0.6070 |
|  | Major | 0.89 (0.36 - 2.23) | 0.8100 |
| **Digestive** | Minor | 1.24 (0.76 - 2.03) | 0.3808 |
|  | Major | 0.28 (0.09 - 0.88) | 0.0295 |
| **Infectious** | Minor | 1.85 (1.29 - 2.65) | 0.0008 |
|  | Major | 1.45 (0.90 - 2.34) | 0.1288 |
| **Hemorrhagic** | Minor | 1.55 (1.24 - 1.93) | 0.0001 |
|  | Major | 1.20 (0.85 - 1.68) | 0.3051 |
| **Urinary tract related** | Minor | 1.07 (0.78 - 1.47) | 0.6684 |
|  | Major | 1.10 (0.77 - 1.58) | 0.5924 |
| **Cardiac** | Minor | 0.89 (0.36 - 2.22) | 0,8030 |
|  | Major | 1.53 (0.69 - 3.38) | 0.2923 |
| **Postoperative VTE**, new events | Minor | 19.58 (17.76 - 21.60) | <.0001 |
|  | Non-minor | 14.57 (12.94 - 16.41) | <.0001 |
| **Outcomes** | **Extent of TURBT (preop-VTE vs. no preop-VTE)** | **aOR** (95%CI) | ***p-value*** |
| **Median costs**, median $ | Minor | 1.23 (1.16 - 1.31) | <.0001 |
|  | Major | 1.07 (0.99 - 1.17) | 0.0888 |
| **Re-hospitalization**, within 90-days | Minor | 1.56 (1.45 - 1.68) | <.0001 |
|  | Major | 1.34 (1.22 - 1.48) | <.0001 |
| **Length of stay**, median days | Minor | 2.11 (1.69 - 2.63) | <.0001 |
|  | Major | 2.43 (1.91 - 3.08) | <.0001 |
| **Discharge status**, other than home | Minor | 1.01 (0.82 - 1.25) | 0.9300 |
|  | Major | 0.90 (0.72 - 1.12) | 0.3241 |
